# Integrative Multi-Transcriptomic Uncovers Actionable Signatures and Drug Repurposing Candidates for ccRCC–Hypertension Comorbidity

**DOI:** 10.3390/cancers18142250

**Published:** 2026-07-14

**Authors:** Yinnan Zhang, Boon Seng Kho, Xixi Wang, Huanhuan Lu, Miao Zhu, Rentao Zhu, Yinyin Wang

**Affiliations:** 1 Department of TCMs Pharmaceuticals, School of Traditional Chinese Pharmacy, China Pharmaceutical University, Nanjing 211198, China; 2 Rehabilitation Department, Changzhou Children’s Hospital, Nantong University, Nantong 213003, China; 3 Department of Hematology, Northern Jiangsu People’s Hospital Affiliated to Yangzhou University, Yangzhou University, Yangzhou 225001, China; 4 Kunshan Fifth People’s Hospital, Suzhou 215341, China

**Keywords:** hypertension, clear cell renal cell carcinoma, integrative analysis

## Abstract

Background: Hypertension is a common comorbidity and risk factor for clear cell renal cell carcinoma (ccRCC), yet the molecular mechanisms linking these two diseases remain unclear. This study aimed to investigate hypertension-related molecular signatures in ccRCC using integrated multi-transcriptomic analyses. Method: Hypertension-related differentially expressed genes (DEHRGs) in ccRCC were identified by integrating transcriptomic data from the TCGA-KIRC cohort with the GeneCards database. Functional enrichment, WGCNA, machine learning, GSVA, survival analysis, and Mendelian randomization were performed to identify and characterize hub genes. Spatial and single-cell transcriptomic analyses were used to investigate tumor heterogeneity and predict candidate therapeutic compounds, which were subsequently evaluated in vitro. Results: Four hypertension-related hub genes—*SCNN1G*, *CASR*, *KCNJ1*, and *WNK4*—were identified and were mainly involved in ion transport, electrolyte homeostasis, and blood pressure regulation. Diagnostic models based on these genes showed good discriminatory performance between tumor and normal tissues, and the nomogram showed good calibration and potential clinical benefit. Higher hypertension-related transcriptional activity was associated with significantly poorer overall survival, while MR analysis suggested a genetic association with ccRCC risk. Spatial transcriptomics revealed pronounced spatial heterogeneity of *SCNN1G*, *CASR*, *KCNJ1*, and *WNK4*, with these hub genes predominantly enriched in stromal and immune regions. Single-cell-based drug screening identified candidate compounds predicted to have antitumor activity, among which epicatechin gallate (ECG) and pseudoginsenoside-F11 showed favorable tumor selectivity. Conclusions: This study systematically characterized hypertension-related molecular signatures in ccRCC and identified *SCNN1G*, *CASR*, *KCNJ1*, and *WNK4* as key hub genes. These genes may contribute to the molecular association between hypertension and ccRCC. They also represent promising candidate biomarkers and therapeutic targets. Integrated spatial and single-cell analyses further identified pseudoginsenoside-F11 and ECG as potential therapeutic compounds and supporting a potential molecular association between hypertension and ccRCC.

## Simple Summary

Clear cell renal cell carcinoma (ccRCC) is the most common subtype of kidney cancer, and hypertension frequently coexists with renal malignancies. However, the molecular mechanisms linking hypertension and ccRCC remain poorly understood. Here, we integrated multi-omics data to identify hypertension-associated molecular features in ccRCC. Four hub genes, *SCNN1G*, *CASR*, *KCNJ1*, and *WNK4*, were identified and were mainly involved in ion transport and blood pressure regulation, suggesting their potential roles in the interplay between hypertension and ccRCC. Spatial transcriptomic analysis further characterized the distribution of these genes within the tumor microenvironment. Single-cell–based drug screening identified potential therapeutic compounds, with epicatechin gallate (ECG) and pseudoginsenoside-F11 showing promising tumor-selective properties. This study provides new insights into the molecular connection between hypertension and ccRCC and highlights potential biomarkers and therapeutic opportunities.

## 1. Introduction

Clear cell renal cell carcinoma (ccRCC) is the most common histological subtype of renal cell carcinoma, accounting for approximately 75% to 80% of cases worldwide [1]. Previous studies have demonstrated that smoking, excessive alcohol consumption, obesity, hypertension, exposure to toxic chemicals such as trichloroethylene, and chronic renal failure are closely associated with the development and progression of ccRCC [2]. In recent years, despite advances in surgery, targeted therapy, and immunotherapy, the overall prognosis of ccRCC remains unsatisfactory. This is mainly due to the lack of specific early clinical symptoms and reliable diagnostic biomarkers, leading to the diagnosis of most patients being diagnosed at an advanced stage [3]. Therefore, elucidating the molecular mechanisms underlying ccRCC and identifying key biomarkers for early diagnosis and prognosis are crucial for improving clinical outcomes.

Among these risk factors, the association between hypertension and ccRCC has attracted increasing attention in recent years. The kidney plays a central role in maintaining blood pressure and electrolyte homeostasis, while dysregulation of hypertension-related molecular networks may directly contribute to renal tumorigenesis and progression [4]. In addition, hypertension is not only a highly prevalent chronic disease but also one of the most common comorbidities in patients with ccRCC [5]. Increasing evidence suggests that long-term hypertension may promote tumor initiation and progression by inducing renal hypoxia, oxidative stress, and tumor microenvironment reprogramming [6,7]. Epidemiological studies have also shown that individuals with hypertension have a significantly higher risk of developing renal cell carcinoma than those with normal blood pressure [8]. On the other hand, certain antihypertensive drugs, particularly those targeting the renin–angiotensin–aldosterone system (RAAS), have also been shown to modulate tumor microenvironment dynamics and ccRCC progression [9]. However, the specific mechanisms by which hypertension-related molecular networks contribute to ccRCC development and progression remain poorly understood.

Notably, most previous studies have focused on clinical correlations or single signalling pathways, while a comprehensive transcriptome-level characterization of hypertension-related gene regulatory networks in ccRCC has not yet been achieved [10]. In recent years, advances in bioinformatics and high-throughput sequencing technologies have provided powerful tools for systematically investigating disease-related molecular mechanisms at multiple molecular levels, as well as for network pharmacology-based drug screening and therapeutic target identification [11]. Recently, multimodal transcriptomic integration and comprehensive herbal medicine resources have further facilitated the elucidation of the mechanisms of action of traditional Chinese medicines and the identification of bioactive compounds and potential combinational therapies through expression-based computational approaches [12,13]. In particular, integrative analytical strategies combined with machine learning algorithms have demonstrated great potential in the identification of tumor biomarkers, disease subtyping, and prognostic evaluation [14]. Among these approaches, Least Absolute Shrinkage and Selection Operator (LASSO) regression and the Random Forest (RF) algorithm have been widely used for feature selection and diagnostic model construction in cancer genomics studies [15,16,17,18,19]. Therefore, this study aimed to systematically identify hypertension-related molecular features in ccRCC using transcriptomic analysis and machine learning approaches, and to develop a candidate diagnostic model, thereby providing new insights into the molecular mechanisms and diagnostic strategies of ccRCC [20,21].

**Figure 1:**
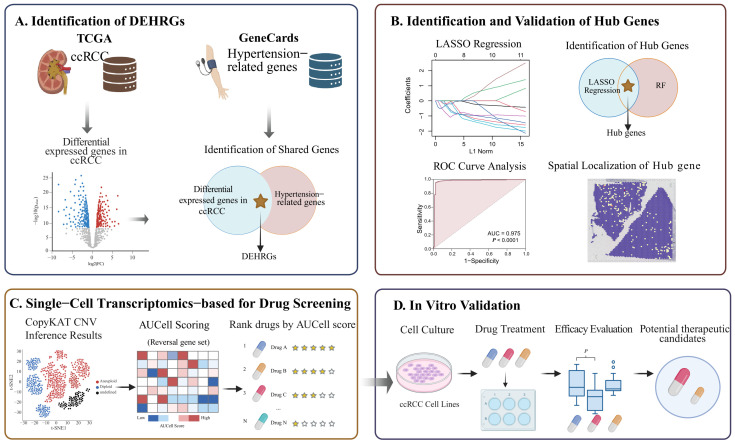
Integrative framework for biomarker discovery, mechanistic analysis, and therapeutic exploration in ccRCC. (**A**) Hypertension-related genes from GeneCards were integrated with TCGA-KIRC data to identify differentially expressed genes. (**B**) LASSO and random forest identified four hub genes. Their diagnostic performance was evaluated by ROC curve analysis, followed by visualization of their spatial expression patterns in ccRCC tissues using spatial transcriptomics. (**C**) A single-cell-based algorithm was used to screen candidate compounds based on AUCell scoring and frug rangking. (**D**) Top drugs were validated in vitro assays in ccRCC cell lines and in silico analyses, including molecular docking and pathway modulation. This integrative approach identifies clinically relevant biomarkers and potential therapeutic targets linking hypertension to ccRCC.

We proposed an integrative multi-transcriptomic approach to uncover actionable signatures and drug-repurposing candidate compounds for the ccRCC–hypertension comorbidity (Figure 1). First, shared differentially expressed genes between ccRCC and hypertension from the Cancer Genome Atlas (TCGA) and GeneCards databases were identified as differentially expressed hypertension-related genes (DEHRGs). Secondly, LASSO regression and RF algorithms were used to identify hub genes from these DEHRGs. Then, the diagnostic performance of hub genes was evaluated using receiver operating characteristic (ROC) curves and a nomogram. Thirdly, to prioritize potential bioactive compounds, we designed a computational method based on single-cell transcriptomic features to prioritize compounds with high enrichment scores on differentially expressed genes of ccRCC.

Overall, by integrating disease-related gene prioritization, machine learning, single-cell transcriptomic analysis, and drug-response prediction within a unified analytical framework, this study provides a comprehensive characterization of hypertension-associated molecular alterations in ccRCC.

## 2. Materials and Methods

### 2.1. Dataset and Process

The hypertension-related genes (HRGs) were retrieved from the GeneCards platform (https://www.genecards.org/) using a relevance score threshold greater than 10 [22]. Transcriptomic expression profiles and corresponding clinical information for clear cell renal cell carcinoma (ccRCC) were obtained from the UCSC Xena platform (https://xena.ucsc.edu/), which comprised 605 samples, including 533 ccRCC tumor samples and 72 solid tissue normal samples. To independently validate the expression levels of the identified hub genes, the GSE53757 dataset was downloaded from the Gene Expression Omnibus (GEO, https://www.ncbi.nlm.nih.gov/geo/; accessed on 21 September 2025), which contains paired ccRCC tumor and adjacent normal kidney tissues. Raw expression data were normalized and log2-transformed as appropriate. Samples with incomplete expression or clinical information were excluded from subsequent analyses. The TCGA-KIRC expression matrix downloaded from UCSC Xena was used directly as provided by the platform. Gene expression values were log2-transformed (log2(x + 1)) before downstream analyses. All data processing steps were performed using R software (version 4.4.2).

### 2.2. Identification and Function Annotation of the Differentially Expressed Hypertension-Related Genes

The differentially expressed genes (DEGs) for ccRCC samples were analysed by applying the “limma” package from R [23]. Genes meeting the criteria of *p* < 0.05 and |log2 fold change| > 3 were defined as DEGs, and a total of 1046 DEGs were screened. The stringent fold-change threshold was applied to retain potentially relevant hypertension-related genes during the initial screening. Next, the differentially expressed hypertension-related genes (DEHRGs) were identified by intersecting the ccRCC DEGs with the HRGs. A total of 27 DEHRGs were screened, as illustrated by using a Venn diagram. Gene Ontology (GO) functional annotation and Kyoto Encyclopaedia of Genes and Genomes (KEGG) pathway enrichment analyses were performed on the DEHRGs using the “clusterProfiler” package [24]. To control the false discovery rate resulting from multiple hypothesis testing, multiple testing correction was performed using the Benjamini–Hochberg (BH) procedure. Enrichment results with adjusted *p* < 0.05 were considered statistically significant. The STRING database (https://string-db.org/) was used to investigate the protein–protein interaction (PPI) network within the DEHRGs [25], with a minimum interaction score of 0.40. The resulting interaction network was visualized to explore potential functional relationships among DEHRGs.

### 2.3. Weighted Gene Co-Expression Network Analysis (WGCNA)

Weighted gene co-expression network analysis (WGCNA) is a computational approach that enables the identification of biologically relevant modules of co-expressed genes and evaluates their association with disease mechanisms. The ccRCC dataset was analyzed using the R package “WGCNA” to determine the optimal soft-threshold power (β), and a soft-thresholding power of 6 was selected to construct the co-expression network [26]. An unsigned network was constructed, and the adjacency matrix was transformed into an unsigned topological overlap matrix (TOM), from which the corresponding dissimilarity matrix (1 − TOM) was calculated. Gene modules were identified using the dynamic tree cut algorithm with a minimum module size of 30 genes. Modules with highly similar eigengenes were merged using a merge cut height of 0.25. Module eigengenes were calculated to summarize the expression profiles of each module, and correlations between module eigengenes and clinical traits were assessed to identify modules most strongly associated with ccRCC. Genes from the most relevant module were retained for downstream analyses.

### 2.4. Machine Learning-Based Identification of Hub Genes

The genes from the key module were intersected with DEHRGs, and the least absolute shrinkage and selection operator (LASSO) and Random Forest (RF) were engaged to screen the intersected genes for their potential diagnostic values.

LASSO regression was performed using the “glmnet” package in R. This regression algorithm applies regularization for variable selection [27]. This algorithm performs penalized regression by adding an L1 regularization term that constrains the sum of absolute coefficients, effectively forcing less informative variables to shrink toward zero. Through 10-fold cross-validation, the optimal penalty parameter (λ) was determined to minimize model error. Genes with non-zero coefficients in the optimal λ model were considered to have significant diagnostic potential. To improve robustness, bootstrap resampling was repeated 100 times, and genes consistently selected across these iterations were defined as stable LASSO-selected features.

The “randomForest” package in R was used to build the RF model [28], RF is a machine learning algorithm commonly used for gene importance analysis. The model was trained with 1000 trees, and mean decrease in accuracy (MDA) and mean decrease in Gini (MDG) were computed to measure the relative importance of each gene. Based on the distribution of RF importance scores, genes with importance scores greater than 6 were retained as candidate genes. The overlapping genes identified by the two algorithms were recognized as hub hypertension-related genes in ccRCC diagnosis.

### 2.5. Diagnostic Model Construction and Survival Analysis

Given that nomogram construction was a crucial metric for clinical ccRCC diagnosis, a diagnostic remodeling model was developed using hypertension-related genes to construct a nomogram via the “rms” package in R [29,30]. Furthermore, to assess the diagnostic ability of these genes in distinguishing between normal and cancer individuals, the receiver operating characteristic (ROC) curve was calculated and plotted [31]. In addition to distinguishing between normal and cancer individuals, Kaplan–Meier analysis was used to evaluate the survival association with DEHGs in ccRCC. Patients were stratified into high- and low-score groups based on the optimal GSVA score on DEGs associated with hypertension phenotypic traits and ccRCC cohorts.

### 2.6. Mendelian Randomization (MR) Analysis

A two-sample Mendelian randomization (MR) framework was applied to investigate the potential causal effect of hypertension-related genetic liability on the risk of clear cell renal cell carcinoma (ccRCC). Summary statistics for hypertension were obtained from the OpenGWAS platform (ID: ieu-b-5144), while ccRCC outcome summary statistics were retrieved from the GWAS Catalog (https://www.ebi.ac.uk/gwas/home; accessed on 25 October 2025). Instrumental variables were selected and harmonized to ensure consistent effect allele orientation. MR analyses were performed using the “TwoSampleMR” R package. The inverse-variance weighted (IVW) method was used as the primary estimator, with MR-Egger, weighted median, simple mode, and weighted mode methods applied as sensitivity analyses.

Potential heterogeneity and pleiotropy were assessed using scatter plots and funnel plots [32]. Furthermore, the causal effects of the hub genes on hypertension were analysed using the inverse-variance-weighted (IVW) method, with two or more available cis-eQTLs per hub gene. The potential causal effects were filtered by *p* < 0.05 as the threshold. This MR analysis was conducted as a complementary layer of genetic inference to support the transcriptomic and machine-learning identification of hypertension-related hub genes (*SCNN1G*, *CASR*, *KCNJ1*, and *WNK4*) in ccRCC.

### 2.7. Spatial Transcriptomic Analysis of the Immune Microenvironment

Spatial transcriptomic data from two ccRCC tumour samples (GSE210041) were obtained from the Gene Expression Omnibus (GEO). Data preprocessing included quality control, normalization, dimensionality reduction, and unsupervised clustering. Spatial domains were annotated using domain-enriched marker genes with reference to the CellMarker database (http://www.bio-bigdata.center/; accessed on 21 January 2026), enabling the evaluation of hub-gene expression across distinct tumour-associated niches. Differential expression and module score analyses were conducted to evaluate spatial expression patterns of hub genes across distinct tumour-associated niches. Visualization was performed using spatial feature plots and violin plots.

### 2.8. Cell Type Deconvolution of Spatial Transcriptomic Data

Kidney cancer–related single-cell RNA sequencing data were used as the reference transcriptomic atlas for cell type deconvolution of spatial transcriptomic profiles. Cell type composition at each spatial spot was inferred using the Robust Cell Type Decomposition (RCTD) algorithm implemented in the R package spacexr [33]. RCTD estimates the relative proportions of distinct cell types within each spatial location by modeling the transcriptional similarity between spot-level expression profiles and reference single-cell transcriptomes, while simultaneously accounting for platform-specific technical effects and differences in transcript count distributions.

For each spatial spot, RCTD generated probabilistic cell type assignments based on maximum likelihood estimation, thereby enabling the identification of potentially mixed cellular compositions across tissue regions. The cell type with the highest inferred proportion was defined as the dominant cell type for subsequent spatial annotation and visualization analyses.

### 2.9. Wet Experimental Validation of the Biomarker Gene

Cell Culture: HK-2 and ACHN cells were kindly provided by Professor Rentao Zhu’s research group from the Department of Urology, Kunshan Fifth People’s Hospital. The 786-O cell line was purchased from Nanjing Runyan Biotechnology Co., Ltd. (Nanjing, China). HK-2 cells were cultured in MEM (Gibco, Thermo Fisher Scientific, Waltham, MA, USA; Cat#C11095500BT) supplemented with 10% FBS (Vazyme Biotech Co., Ltd., Nanjing, China; Cat#7E970D5) and 1% penicillin-streptomycin (Solarbio Life Sciences, Beijing, China; Cat#P1400). ACHN and 786-O cells were maintained in RPMI-1640 medium (Gibco, Cat#C11875500BT) containing 10% FBS (Vazyme, Cat#7E970D5) and 1% penicillin-streptomycin (Solarbio, Cat#P1400). All cells were cultured in a humidified incubator at 37 °C with 5% CO_2_. Cells were passaged every 1–2 days on average, and fungal contamination was tested once per week.

Plasmid Transfection: *SCNN1G* knockdown plasmids were constructed by Punoen Biotechnology Co., Ltd. (Nanjing, China). The shRNA sequences were as follows:

- shRNA1:
- ACCGGCAGACTTGGCCAAACTCTTCTCGAGAAGAGTTTGGCCAAGTCTGTTTTTTGAATTC.
- shRNA2:
- ACCGGCCGACCATTAAAGAGCTGACTCGAGTCAGCTCTTTAATGGTCGGTTTTTTGAATTC.
- shRNA3:
- ACCGGCGATGCATGGGAATTGCTACTCGAGTAGCAATTCCCATGCATCGTTTTTTGAATTC.

Cells were seeded into 24-well plates 24 h prior to transfection. When cell confluence reached approximately 80%, the medium was replaced with fresh antibiotic-free medium. The transfection complex was prepared by mixing 2 μg plasmid DNA with 200 μL serum-free medium and 4 μL Lipofectamine 3000 (Thermo Fisher Scientific, Waltham, MA, USA; Cat#L3000001), followed by incubation at room temperature for 20 min. The complexes were then added to the cells, which were returned to the incubator. Transfection efficiency was assessed by fluorescence microscopy after 24–48 h.

Immunofluorescence Assay: Sterilized coverslips placed in 6-well plates were seeded with logarithmically growing cells. Upon reaching proximately 60% confluence, the coverslips were collected, and the cells were fixed with 4% paraformaldehyde for 20 min at room temperature. Following three PBS washes, cells were permeabilized with 0.20% Triton X-100 for 15 min, washed again, and blocked with 5% BSA for 30 min. The blocking solution was then replaced with *SCNN1G* primary antibody (AffinityBiosciences, Changzhou, China; Cat#DF8540) diluted 1:100, and incubation proceeded overnight at 4 °C in the dark. After additional PBS washes, cells were exposed to fluorescent secondary antibody (Invitrogen, Thermo Fisher Scientific, Waltham, MA, USA; Cat#A56580) diluted 1:100 for 2 h at 37 °C in the dark. Following final PBS rinses, coverslips were mounted with DAPI-containing medium and air-dried in the dark for 30 min at room temperature. Fluorescence microscopy was used for image acquisition.

qRT-PCR: Total RNA was extracted using a spin-column kit (NCM Biotech, Suzhou, China; Cat#M5105) per the manufacturer’s protocol, encompassing lysis, washing, and elution. RNA concentration was measured, and 1 μg of total RNA was reverse-transcribed with a TAKARA kit (Takara Bio Inc., Kusatsu, Japan; Cat#RR047A). The resulting cDNA served as template for qPCR reactions containing 0.5 μL each of forward and reverse primers, 2 μL cDNA, 2 μL ddH_2_O, and 5 μL SYBR Green Mix (Applied Biological Materials Inc. (ABM), Richmond, BC, Canada;, Cat#MasterMix-S). Relative expression was calculated via the $2^{-\Delta \Delta C_{T}}$ method. Primer sequences were as follows:

- *SCNN1G* F: GAGTGACGTGCCAATCAGGA
- *SCNN1G* R: TCTCCGAAACCACAGATGGC
- β-actin F: GCACTCTTCCAGCCTTCCTTCC
- β-actin R: GCGGATGTCCACGTCACACTTC

Western Blot: Harvested cells were washed twice with PBS and lysed in RIPA buffer on ice for 20 min. Lysates were scraped, collected, and centrifuged at 12,000× *g* for 15 min at 4 °C. The supernatant was retained, and protein concentrations were determined via BCA assay to ensure equal loading. Proteins were resolved by SDS-PAGE, transferred to membranes, and blocked with 5% non-fat milk for 2 h at room temperature. Membranes were then incubated with anti-SCNN1G primary antibody (1:1000) overnight at 4 °C with gentle agitation. After three 10 min TBST washes, membranes were treated with secondary antibody for 2 h at room temperature, washed again with TBST, and developed using ECL reagents for chemiluminescent detection.

Colony Formation Assay: Log-phase cells were trypsinized and resuspended as single cells. After counting, 1000 cells per well were seeded into 6-well plates and cultured for 10–14 days with regular monitoring. Once visible colonies emerged, the medium was aspirated, and cells were washed twice with PBS, fixed with 4% paraformaldehyde for 15 min, and stained with 0.5% crystal violet for 20 min. Following rinsing and drying, colonies were imaged and quantified using Fiji software (v2.14.0).

Apoptosis Assay: Cells and their corresponding culture supernatants were pooled and centrifuged at 1200 rpm for 5 min at room temperature. The pellet was resuspended in 400 μL binding buffer, followed by the addition of 5 μL Annexin V-APC and 5 μL 7-AAD. After a 20 min dark incubation, samples were analyzed by flow cytometry within 1 h, with data processed via FlowJo software (v10.8.1).

Transwell Assay: Cells were trypsinized and suspended in serum-free medium. After counting, 2 × 10^4^ cells in 200 μL were placed into the upper Transwell chambers, while 800 μL of complete medium was added to the lower chambers. Following 24 h of incubation, the inserts were removed; non-migratory cells on the upper surface were gently wiped off, while cells on the lower surface were fixed with 4% paraformaldehyde for 15 min and stained with 0.5% crystal violet for 20 min. After washing and air-drying, images were captured and migrated cells were enumerated with Fiji.

Wound Healing Assay: Cells seeded in 6-well plates were grown to >90% confluence. A straight scratch was created in each well using a 1 mL pipette tip, and detached cells were removed by gentle PBS washing. Images were taken immediately (0 h), then again at the same positions after 24 h in serum-free medium. Wound closure was assessed by measuring scratch widths with Fiji software, and migration percentage was calculated accordingly.

High-Content Cell Tracking Assay: Cells were seeded into black-walled, clear-bottom 96-well plates. After resuspension and counting, 2000 cells per well were plated. Once cells adhered, the plate was placed into a high-content imaging system. Nine fields per well were selected, and images were acquired every 10 min for a total duration of 10 h. Cell movement and related parameters were analyzed using the accompanying software.

### 2.10. Construction of Disease and Drug Transcriptional Signatures and Identification of Candidate Drugs Based on Expression Reversal

An integrative algorithm was developed to prioritize compounds with tumor-selective reversal potential by quantifying their ability to reverse disease-associated transcriptional programs in tumor cells while minimizing effects in normal cells. ccRCC disease signatures were derived from single-cell RNA-seq data. CopyKAT was applied to infer copy number variations and classify cells into aneuploid tumor and diploid normal populations [34]. Differential expression analysis between these groups identified significantly dysregulated genes, which were defined as disease-associated transcriptional signatures.

Drug-induced transcriptional profiles were obtained from two sources. L1000 small-molecule signatures were retrieved from the LINCS database using consensus perturbation profiles. For traditional Chinese medicine (TCM), transcriptional data from the ITCM database and published datasets were processed, normalized, and subjected to differential expression analysis (|log2FC| > 0.585, *p* < 0.05) to define up- and downregulated gene sets.

Drug screening was performed by evaluating the directional reversal between disease and drug signatures. AUCell was used to quantify gene set activity at the single-cell level, and scores were aggregated across tumor and normal cells to assess tumor-selective reversal efficacy. The overall drug score was defined as follows:

$$S_{drug}=\frac{1}{n_{t}}\sum_{i=1}^{n_{t}}A_{i}^{tumor}-\frac{1}{n_{n}}\sum_{j=1}^{n_{n}}A_{j}^{normal}$$

where $A_{i}^{tumor}$ denotes the AUCell score of the reversal gene set in the *i*-th aneuploid tumor cell, and $A_{j}^{normal}$ denotes the AUCell score in the *j*-th diploid normal cell. $n_{t}$ and $n_{n}$ represent the numbers of tumor and normal cells, respectively.

### 2.11. Validation of Potential Bioactive Compounds Using theCCK-8 Assay

Logarithmically growing cells were trypsinized and resuspended as a single-cell suspension. After counting, 2000 cells in 100 μL of culture medium were seeded per well into 96-well plates and incubated overnight at 37 °C in a 5% CO_2_ atmosphere. The next day, the old medium was aspirated and replaced with fresh medium containing graded concentrations of the test compound; blank wells (medium only, no cells) were also prepared. Following 24 h of treatment, 10 μL of CCK-8 reagent was added to each well, and plates were incubated for an additional 4 h. Absorbance (OD) was recorded using a microplate reader. Cell viability was calculated as: Viability (%) = (OD_test − OD_blank)/(OD_0μM − OD_blank) × 100%, and dose–response curves were plotted accordingly.

### 2.12. Statistical Analysis

All data were analyzed using GraphPad Prism 10.0 software and R (version 4.4.2). Experiments were performed independently at least three times, and results are presented as mean ± standard deviation (SD). Comparisons between two groups were performed using a two-tailed Student’s *t*-test, while multiple group comparisons were conducted using one-way ANOVA. *p* < 0.05 was considered statistically significant (* *p* < 0.05, ** *p* < 0.01, *** *p* < 0.001).

## 3. Results

### 3.1. Systematic Screening and Functional Characterization of the Hypertension-Related Hub Genes in ccRCC

To systematically investigate the roles of hypertension-related genes (HRGs) in ccRCC, an integrated analytical framework incorporating differential expression analysis, network module analysis, and cross-validation strategies was employed to identify key HRGs associated with ccRCC.

**Figure 2:**
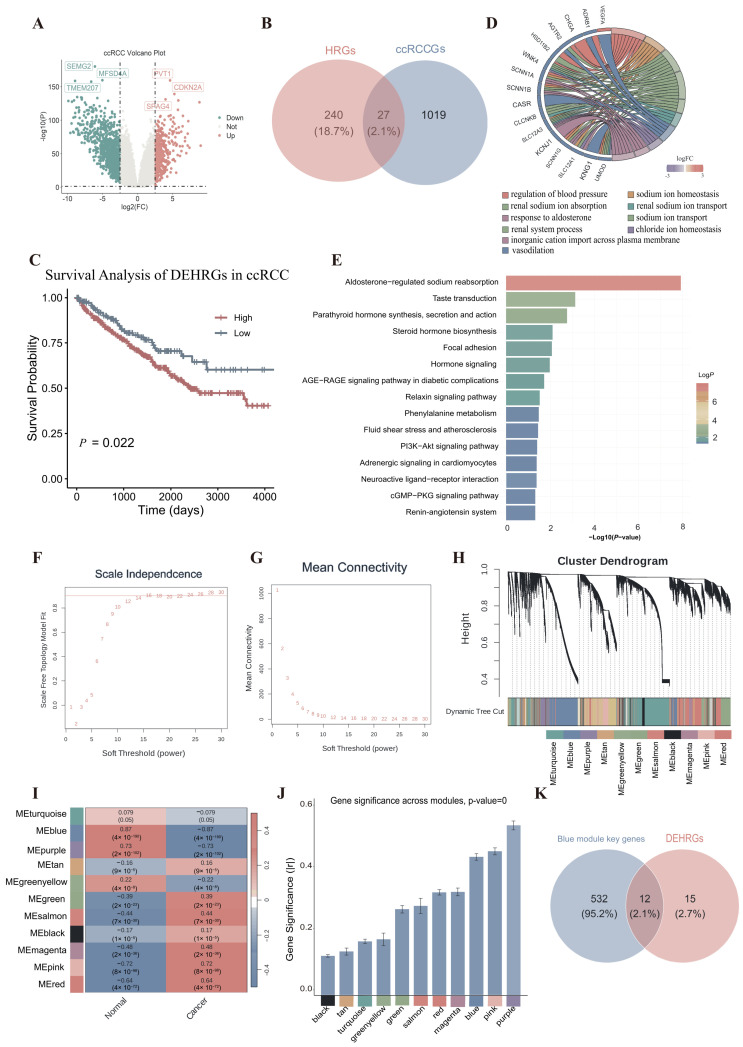
Identification and enrichment analyses of DEHRGs. (**A**) Volcano plot for ccRCC DEGs. (**B**) Screening of common genes between HRGs and ccRCCGs. (**C**) Kaplan–Meier survival analysis showing the prognosis of ccRCC patients categorized by DEHRGs expression. (**D**) GO enrichment for DEHRGs. (**E**) KEGG pathway enrichment analyses for DEHRGs. (**F**) The determination of soft thresholding power β in the WGCNA. (**G**) The mean connectivity across different soft threshold powers. (**H**) Hierarchical clustering dendrogram of genes. (**I**) Module-trait relationship, as the blue module was significantly associated with clear cell renal cell carcinoma (ccRCC). (**J**) Distribution of mean gene significance across different modules associated with ccRCC. (**K**) The Venn diagram shows that twelve common genes are identified at the intersection of the blue module and DEHRGs.

Initially, a total of 267 HRGs were retrieved from the GeneCards database using a relevance score ≥ 10 as the screening criterion. Subsequently, differential expression analysis was performed on 607 ccRCC samples, including 534 tumor samples and 73 normal samples. Using thresholds of *p* < 0.05 and |log2FC| value > 3, a total of 1046 ccRCC-associated DEGs (ccRCCGs) were identified (Figure 2A). Intersection analysis between ccRCCGs and HRGs identified 27 differentially expressed hypertension-related genes (DEHRGs) (Figure 2B), suggesting a potential association between hypertension-related biological processes and ccRCC.

To further evaluate the association between hypertension-related transcriptional gene signatures and patient prognosis, Gene set variation analysis (GSVA) was performed to assess the transcriptional activity of DEHRGs in ccRCC patients. Patients were subsequently stratified into high- and low-score groups based on the optimal GSVA score cutoff. Kaplan–Meier survival analysis demonstrated that patients with higher GSVA scores exhibited significantly reduced overall survival (log-rank *p* = 0.02) (Figure 2C), indicating that hypertension-related transcriptional activity was associated with poor prognosis in ccRCC.

Following the identification of DEHRGs, GO and KEGG enrichment analyses were performed to further explore their biological functions and underlying mechanisms. GO analysis revealed that DEHRGs were primarily involved in biological processes related to blood pressure regulation, sodium and chloride ion transport, renal system processes, and aldosterone response (Figure 2D). KEGG analysis further demonstrated that DEHRGs were significantly enriched in pathways associate with aldosterone-regulated sodium reabsorption, hormone signalling pathways, focal adhesion, and AGE–RAGE signalling pathway (Figure 2E). In addition, protein–protein interaction (PPI) analysis revealed extensive functional interactions among the DEHRGs, suggesting their coordinated involvement in the regulation of renal ion homeostasis and blood-pressure-related molecular networks (Figure S1).

Building on these findings, weighted gene co-expression network analysis (WGCNA) was further performed to validate the candidate genes at the network level and to identify ccRCC-associated co-expression modules. Notably, DEHRGs identified by differential expression analysis could not fully reflect the global co-expression architecture of the transcriptome. Therefore, WGCNA was applied to incorporate whole-transcriptome co-expression information and further validate candidate genes at the network level. A co-expression network was generated using the expression profiles of all genes. A soft thresholding power of 12 was selected to ensure that the resulting network conformed to a scale-free topology (Figure 2F,G). This analysis identified 11 distinct gene modules (Figure 2H). Module–trait relationship analysis revealed that the blue module (MEblue) exhibited the strongest negative correlation with ccRCC (cor = −0.87, *p* = 4 × 10^−190^), whereas the pink module showed the strongest positive correlation with ccRCC (cor = 0.72, *p* = 8 × 10^−98^) (Figure 2I). Based on module-trait correlations and the distribution of gene significance (GS), the blue module was selected as the key module for subsequent analyses (Figure 2J). Notably, although MEblue showed the strongest correlation with the tumor phenotype, its average gene significance (|GS|) was not the highest among all modules, suggesting the presence of intramodular heterogeneity, and heterogeneous contributions of individual genes to the module–phenotype association.

To further improve the robustness of candidate gene screening, an integrative strategy combining differential expression analysis with co-expression module analysis was employed for cross-validation. A total of 544 genes from the MEblue were intersected with the previously identified 27 DEHRGs, resulting in 12 overlapping genes: *HSD11B2*, *WNK4*, *SCNN1B*, *SCNN1G*, *SCNN1A*, *UMOD*, *BMPR1B*, *KNG1*, *CLCNKB*, *SLC12A1*, *CASR*, and *KCNJ1* (Figure 2K).

Integrative analysis combining differential expression and WGCNA-based network analysis identified a set of hypertension-related core genes closely associated with ccRCC, supporting subsequent mechanistic and prognostic analyses.

### 3.2. Identification of Hypertension-Related Hub Genes in ccRCC Using Machine Learning and Multi-Level Validation

To further identify robust hub genes from the candidate DEHRGs, machine learning analyses using LASSO regression and RF algorithms were performed.

**Figure 3:**
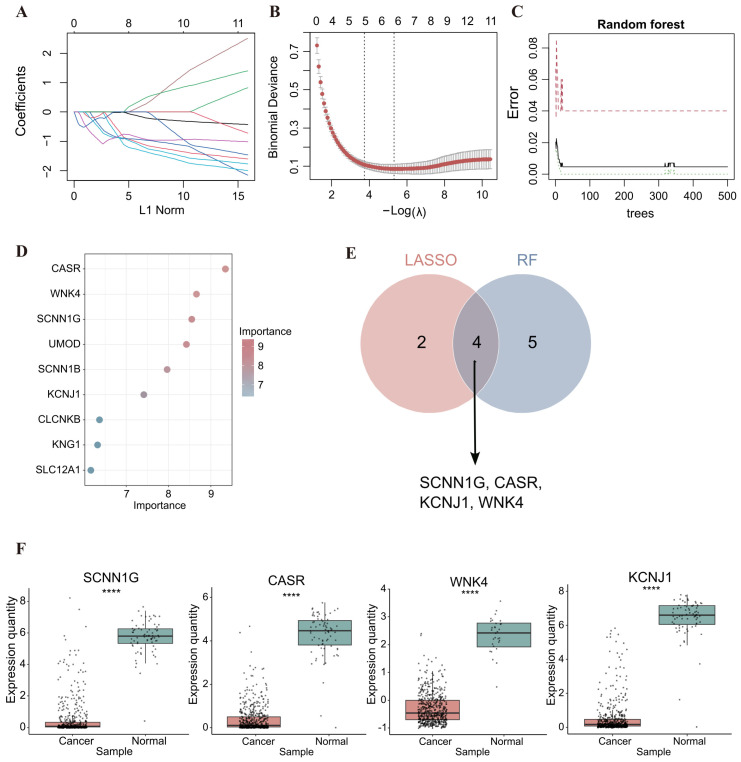
Machine learning analysis to screen biomarkers for diagnosing ccRCC. (**A**,**B**) The LASSO regression revealed that the number of genes corresponding to the lowest point of the curve (*n* = 6) is the optimal value for diagnosing ccRCC. (**C**,**D**) The random forest algorithm showed errors in ccRCC; each gene is ranked by its importance score. (**E**) The Venn diagram illustrates the intersection between genes of LASSO and RF models. (**F**) The expression of four hub genes (*SCNN1G*, *CASR*, *KCNJ1*, *WNK4*) was shown by boxplots. (**** *p* < 0.001).

First, LASSO regression analysis was applied for dimensionality reduction and feature selection, resulting in the identification of six feature genes (Figure 3A,B). Meanwhile, the RF algorithm was employed to rank genes according to their importance score, and genes with importance scores > 6 were selected as the candidate genes (Figure 3C,D). Intersection analysis between the LASSO and RF results identified the hypertension-related hub genes: *SCNN1G*, *CASR*, *KCNJ1*, and *WNK4* (Figure 3E). Further expression analysis demonstrated that all four hub genes were significantly downregulated in ccRCC tissues (*p* < 0.001), suggesting their potential involvement in ccRCC progression (Figure 3F, Supplementary Table S1). To further validate the robustness of the above findings, we performed external validation of the expression levels of the four key genes in an independent renal cell carcinoma cohort (GSE53757). The results showed that all four genes showed significantly higher expression in ccRCC tumor tissues compared with solid tissue normal (Figure S2A), highly consistent with those observed in the TCGA-KIRC cohort.

**Figure 4:**
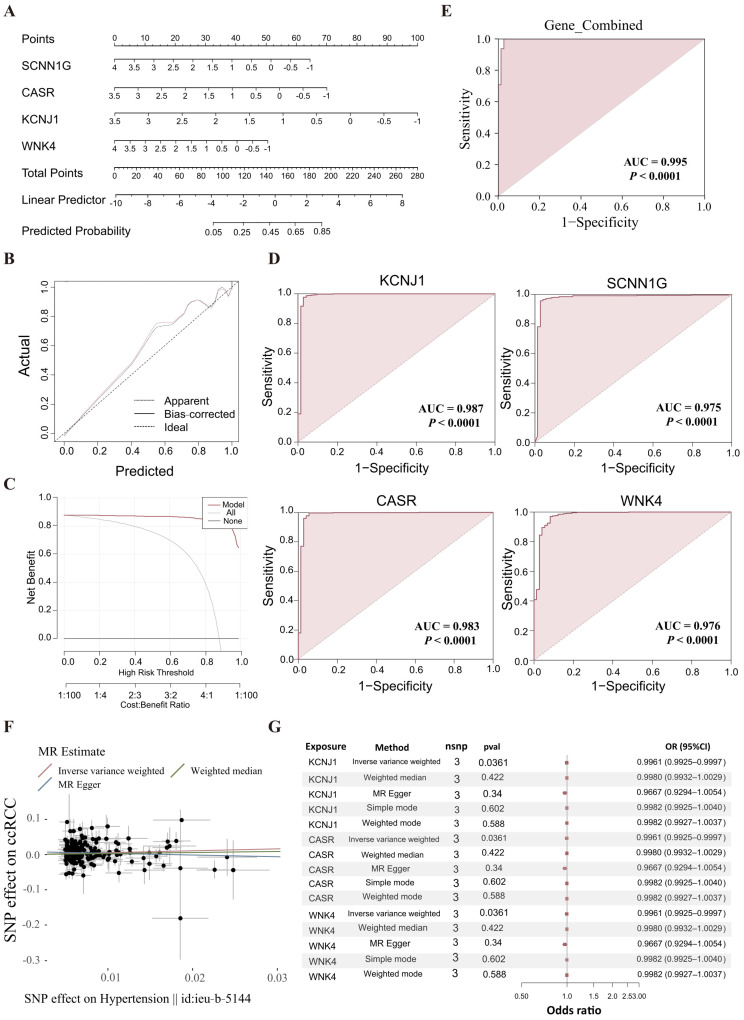
Construction of a nomogram model and assessment of diagnostic value. (**A**) Construction of the nomogram based on four hub hypertension-related genes. (**B**) The calibration curve evaluates the predictive accuracy of the nomogram model. (**C**) The DCA curve assesses the clinical application value of the nomogram model. (**D**) The ROC plots for the hub genes (*SCNN1G*, *KCNJ1*, *CASR*, *WNK4*) indicate the potential diagnostic value. (**E**) ROC curve of the four-gene combined diagnostic model for distinguishing ccRCC from normal kidney tissues. (**F**) Two-sample MR scatter plot showing the estimated causal effect using IVW, MR−Egger, and weighted median methods. (**G**) Forest plot summarizing MR effects estimates (OR and 95% CI) of hub genes across various MR methods.

Then, the nomogram model was developed to assess how diagnostic value changed with clinical survival of these hub genes (Figure 4A). Calibration curve analysis demonstrated good agreement between the predicted and observed values (Figure 4B). In addition, this model shows favorable clinical net benefit across a wide range of threshold probabilities according to the decision curve analysis (DCA) (Figure 4C). Diagnostic performance of these hub genes was further confirmed by their AUC values, with *SCNN1G*, *KCNJ1*, *WNK4*, and *CASR* as 0.975,0.987, 0.976, and 0.983, respectively (Figure 4D). In addition, the combined four-gene model achieved an AUC of 0.995 (Figure 4E), suggesting an improved diagnostic performance compared with individual genes. By comparison, models incorporating gender or age alone showed limited diagnostic value, with AUCs of 0.537 and 0.538 (Figure S2B,C).

To further explore the association of the hub genes with tumor progression, ROC analyses were performed to evaluate their ability to discriminate pathological stage and histological grade. For pathological stage (Stage I–II vs. Stage III–IV), *SCNN1G*, *KCNJ1*, *WNK4*, and *CASR* achieved AUC values of 0.624, 0.531, 0.528, and 0.585, respectively, while the combined four-gene model yielded an AUC of 0.664 (Figure S2E). For histological grade (Grade 1–2 vs. Grade 3–4), the AUC values of *SCNN1G*, *KCNJ1*, *WNK4*, and *CASR* were 0.564, 0.556, 0.521, and 0.564, respectively, and the combined model achieved an AUC of 0.618 (Figure S2F). These results suggest that although the four-gene signature exhibited outstanding diagnostic performance, it showed only modest discriminatory ability for pathological stage and histological grade, with the combined model consistently outperforming the individual genes.

To investigate the potential genetic associations between the hub genes and ccRCC, two-sample MR analysis was performed. Scatter plot analysis demonstrated that the effect directions estimated by different MR methods were generally consistent (Figure 4F), indicating overall concordance among the MR approaches. In the forest plot summary (Figure 4G), the IVW estimator yielded a nominally significant association (*p* = 0.0361), with an OR of 0.9961 (95% CI: 0.9925–0.9997). However, the weighted median, MR-Egger, and other robust MR methods did not reach statistical significance. Given that only three SNP instruments were included in the analysis, these findings should be interpreted as supportive rather than definitive evidence of a causal relationship.

Taken together, machine learning-based screening, clinical predictive model construction, and integrated genetic analyses systematically identified four robust hypertension-related hub genes (*SCNN1G*, *CASR*, *KCNJ1*, and *WNK4*). These genes were significantly downregulated in ccRCC and exhibited robust diagnostic and predictive value, indicating their potential role as molecular mediators linking hypertension and ccRCC.

### 3.3. Spatial Transcriptomics Reveals the Heterogeneity of Hypertension-Related Hub Genes in ccRCC

To characterize the spatial distribution and transcriptional characteristics of hypertension-related genes in ccRCC tissues, spatial transcriptomic analysis was performed using the GSE210041 dataset from the Gene Expression Omnibus (GEO) database.

Quality control was performed on the spatial transcriptomic data, and low-quality spots were filtered out. The expression matrix was subsequently normalized to ensure comparability across spots for downstream analyses. Spatial functional analysis was performed based on the four identified hypertension-related hub genes (*SCNN1G*, *CASR*, *KCNJ1*, and *WNK4*) and DEHRGs to investigate their spatial expression patterns in ccRCC tissues.

**Figure 5:**
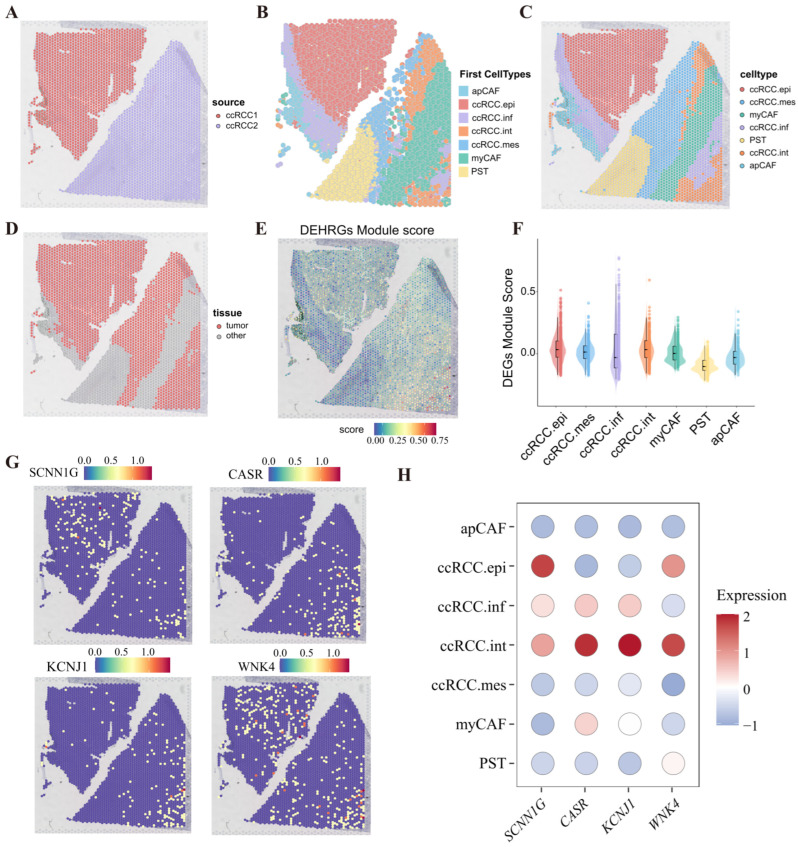
Spatial transcriptomics reveals the heterogeneity of hypertension-related hub genes in ccRCC. (**A**) Spatial transcriptomic maps of different samples. (**B**) Pie chart showing the cell type proportions inferred by RCTD. (**C**) RCTD-based dominant cell type annotation of each spatial spot. (**D**) Spatial segmentation of the ccRCC core tumor region. (**E**,**F**) The spatial distribution and violin plot illustrated the enrichment of a specific cluster by the hub genes. (**G**) Spatial features plots showing the expression patterns of *SCNN1G*, *CASR*, *KCNJ1*, and *WNK4*. (**H**) Dot plot showing the scaled average expression levels of *SCNN1G*, *CASR*, *KCNJ1*, and *WNK4* across different cell populations. Dot color represents the scaled average expression level (Z-score) of each gene across cell types, with red indicating relatively higher expression and blue indicating relatively lower expression.

Based on spatial expression profiles and histological architecture, single-cell transcriptomic data were used as the reference expression atlas, and the Robust Cell Type Decomposition (RCTD) algorithm was applied to infer the cellular composition of each spatial location (Figure 5A,B). The dominant cell type with the highest proportion within each spot was defined as the primary cell type of that spot. Through this approach, seven distinct functional regions were identified, including epithelial-like tumor cells (ccRCC.epi), mesenchymal-like tumor cells (ccRCC.mes), inflammatory-like tumor cells (ccRCC.inf), intermediate-state tumor cells (ccRCC.int), myofibroblastic CAFs (myCAFs) and antigen-presenting CAFs (apCAFs), as well as proximal straight tubule (PST) cells (Figure 5C). These results highlighted marked spatial heterogeneity within the ccRCC tumor microenvironment.

To further evaluate the spatial activity of the hypertension-related molecular signature, a DEHRG-based gene signature was defined and module scores were calculated for each spatial spot. The results revealed significant differences in hypertension-related signature scores across distinct regions (Figure 5D–F). Hypertension-related transcriptional signatures were predominantly elevated in stromal and immune-infiltrated regions, whereas relatively lower scores were observed in tumor epithelial regions (Figure 5F). These findings indicate that hypertension-related transcriptional activity is predominantly associated with the tumor microenvironment rather than being restricted to tumor epithelial cells.

The spatial expression patterns of the four key hub genes (*SCNN1G*, *CASR*, *KCNJ1*, and *WNK4*) were examined. All four genes exhibited distinct spatial expression patterns across different regions (Figure 5G). *SCNN1G* was predominantly expressed in ccRCC.epi cells, whereas *CASR*, *KCNJ1*, and *WNK4* were mainly enriched in ccRCC.int cells (Figure 5H). Notably, all four genes were detected in ccRCC-associated malignant cell states rather than being exclusively confined to normal tubular structures. Given that ccRCC.int represents an intermediate malignant cell state, the preferential enrichment of *CASR*, *KCNJ1*, and *WNK4* in this population suggests that these genes may be associated with the transcriptional programs accompanying the transition from normal renal tubular epithelial cells to malignant ccRCC cells, rather than simply reflecting differences in tumor purity or tissue composition.

Spatial transcriptomic analysis revealed pronounced spatial heterogeneity in ccRCC tissues. Hypertension-related transcriptional signatures were predominantly localized in stromal and immune-associated regions, suggesting their potential involvement in tumor microenvironment regulation in ccRCC.

### 3.4. In Vitro Functional Characterization of SCNN1G in RCC

As a key regulatory gene of the epithelial sodium channel, *SCNN1G* plays a critical role in ion transport and the maintenance of blood pressure homeostasis. Based on a review of the available literature, *SCNN1G* has the most extensively characterized role in hypertension among the four identified hub genes, with substantial evidence supporting its involvement in sodium handling and blood pressure regulation [35,36]. In addition, among the four hub genes, *SCNN1G* exhibited the highest predictive performance for pathological stage (Figure S2E). Combined with its aberrant expression pattern, favorable diagnostic and prognostic performance, consistent findings across our integrated transcriptomic analyses, and its association with pathological stage, *SCNN1G* was selected as the representative hub gene for subsequent experimental validation to further explore the potential link between hypertension-related ion homeostasis dysregulation and ccRCC progression. Although *CASR*, *KCNJ1*, and *WNK4* have also been implicated in blood pressure regulation, their roles in hypertension are comparatively less well characterized, and experimental validation of these genes was therefore beyond the scope of the present study. Their biological functions and molecular mechanisms in ccRCC warrant further investigation in future studies. To investigate the role of *SCNN1G* in renal cell carcinoma (RCC), we selected the human ccRCC cell line 786-O and the human renal adenocarcinoma cell line ACHN for in vitro experiments, using the human proximal tubular epithelial cell line HK-2 as a negative control.

**Figure 6:**
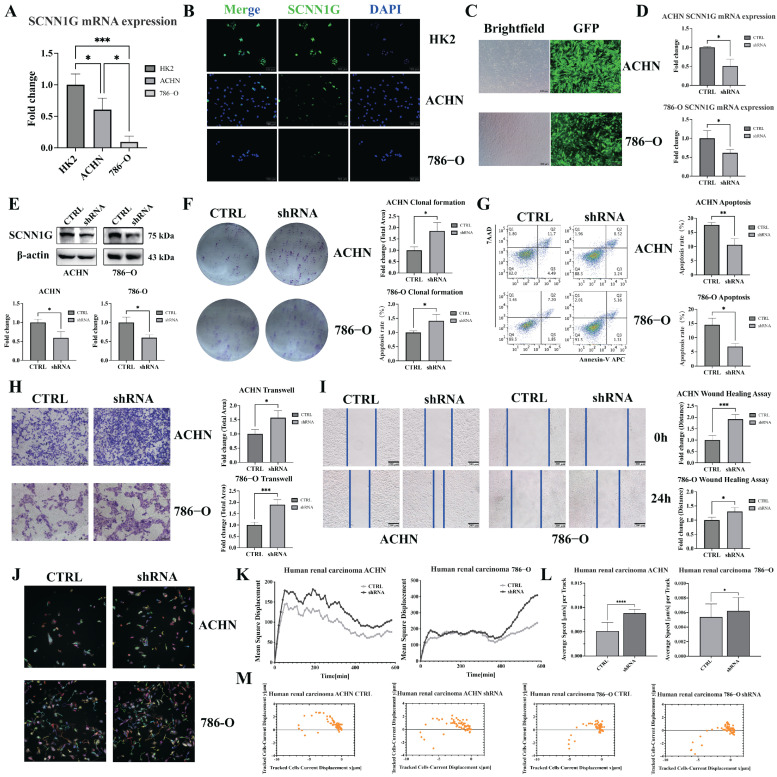
Functional validation of *SCNN1G*. (**A**) qRT−PCR indicates that *SCNN1G* expression is significantly higher in HK−2 cells than in 786−O and ACHN cells. (**B**) SCNN1G protein is highly expressed in both the plasma membrane and nucleocytoplasm of renal tubular epithelial cells, but shows low expression in the nucleocytoplasm of renal cancer cells. Scale bar = 100 μm. (**C**) RCC cells exhibit green fluorescence following *SCNN1G* knockdown. (**D**) qRT−PCR confirms the efficiency of *SCNN1G* knockdown. (**E**) Western blot analysis shows reduced SCNN1G protein levels in the knockdown group. (**F**) Colony formation assays demonstrate enhanced proliferative capacity after *SCNN1G* knockdown. (**G**) Apoptosis levels are decreased in the knockdown group. (**H**,**I**) Transwell and wound healing assays reveal that *SCNN1G* knockdown enhances the migratory ability of RCC cells. (**J**) Representative trajectories of cell movement over 10 h in control and knockdown groups. (**K**,**L**) Quantitative analysis of migration distance and velocity in control and knockdown groups. (**M**) Spatial distribution of cell movement over 10 h in control and knockdown groups. Data are presented as the mean ± SD from at least three independent experiments. Comparisons between two groups were analyzed using an unpaired two-tailed Student’s *t*-test, whereas comparisons among multiple groups were performed using one−way ANOVA. Statistical significance was defined as * *p* < 0.05, ** *p* < 0.01, *** *p* < 0.001, and **** *p* < 0.0001.

qRT-PCR analysis demonstrated that *SCNN1G* expression was significantly higher in HK-2 cells compared with 786-O and ACHN cells (Figure 6A), indicating downregulation of *SCNN1G* in renal cancer cell lines. Immunofluorescence analysis further revealed that in HK-2 cells, *SCNN1G* was predominantly localized to the plasma membrane and cytoplasm, whereas its expression was markedly reduced in RCC cell lines and mainly restricted to the cytoplasmic compartment (Figure 6B). These findings suggest that *SCNN1G* may be dysregulated in RCC and may potentially exert tumor-suppressive functions.

Subsequently, *SCNN1G* expression was silenced in both RCC cell lines by transfection with an *SCNN1G* −targeting shRNA plasmid. Following transfection, green fluorescence signals confirmed successful transfection efficiency (Figure 6C). qRT −PCR analysis demonstrated efficient knockdown of *SCNN1G* expression (Figure 6D), which was further validated by Western blot analysis showing a marked reduction in SCNN1G protein levels (Figure 6E and Figure S3).

Functional assays demonstrated that *SCNN1G* knockdown significantly enhanced RCC cells proliferation (Figure 6F), while markedly reducing apoptosis (Figure 6G). Meanwhile, Transwell and wound healing assays revealed that *SCNN1G* silencing significantly increased the migratory and invasive abilities of RCC cells (Figure 6H,I).

Furthermore, time-lapse imaging over a 10 h period revealed an expanded migration range in the *SCNN1G* knockdown group (Figure 6J). Quantitative analysis confirmed that both migration distance and velocity were significantly increased following *SCNN1G* silencing (Figure 6K,L). In addition, the spatial pattern of cell motility was markedly altered (Figure 6M).

Collectively, these results indicate that *SCNN1G* functions as a tumor suppressor in RCC. Its downregulation promotes RCC cell proliferation, migration, and invasion, suggesting its potential as a therapeutic target for ccRCC.

### 3.5. Identification of Candidate Therapeutic Compounds for ccRCC Based on Single-Cell Transcriptomic Analysis

**Figure 7:**
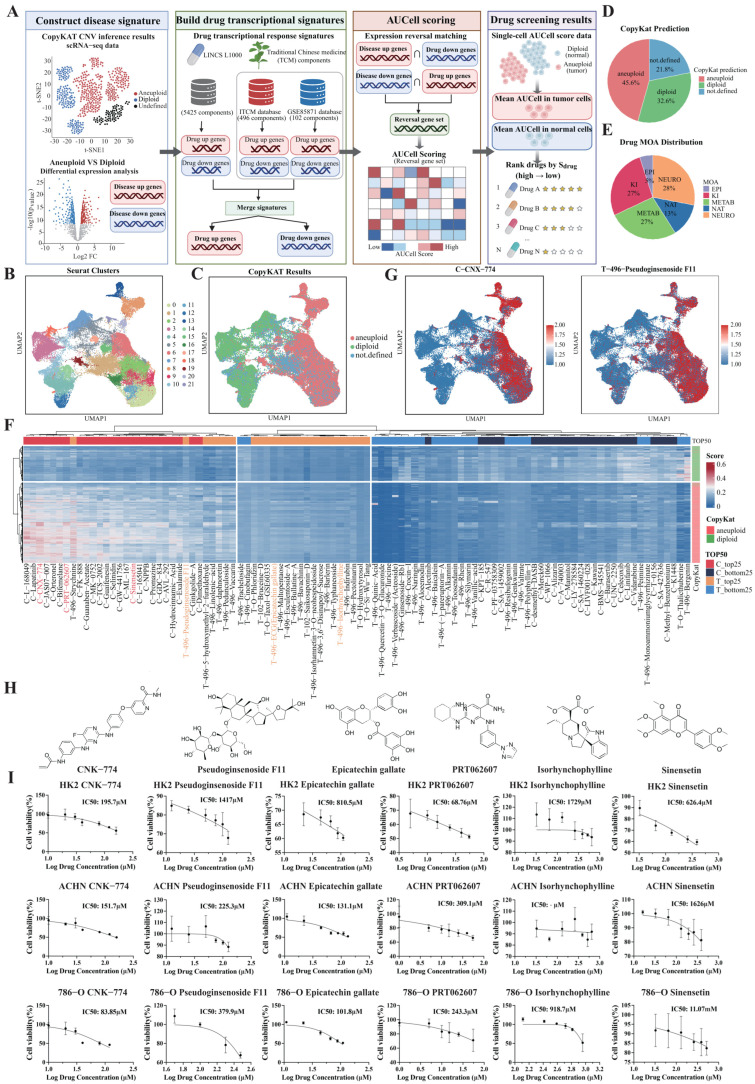
Screening the bioactive ingredients for ccRCC. (**A**) Schematic overview of the drug screening pipeline. (**B**) UMAP visualization of Seurat-defined cell clusters in ccRCC single-cell RNA-seq data. (**C**) UMAP plot of copyKAT-inferred aneuploid and diploid cells states. (**D**) Pie chart showing the proportions of copyKAT-inferred aneuploid and diploid cells. (**E**) Pie chart showing the distribution of mechanisms of action of the top 100 predicted drugs. (**F**) Clustered heatmap of the top 25 and bottom 25 LINCS L1000 database and TCM compound library identified in the drug screening analysis. (**G**) UMAP visualization of drug screening scores in single-cell RNA-seq data. (**H**) Chemical structures of the screened drug candidates. (**I**) The IC50 values of the selected compounds were determined in renal tubular epithelial cells and renal carcinoma cells.

To explore potential therapeutic strategies for ccRCC, a single-cell transcriptomics-based drug screening framework was established, and candidate compounds were systematically prioritized according to hypertension-related transcriptional signatures (Figure 7A).

Dimensionality reduction and clustering analyses were first performed on the ccRCC single-cell transcriptomic dataset (Figure 7B). Subsequently, CopyKAT analysis was applied to infer copy number variation (CNV) profiles at the single-cell level, enabling discrimination between aneuploid and euploid cell populations (Figure 7C,D). Based on these analyses, a drug response scoring system was established using disease-associated transcriptional signatures, and AUCell analysis was performed to quantify drug response activity across distinct cellular populations (Figure 7A). Candidate compounds were subsequently systematically ranked according to their drug response scores. Among the top 100 candidate compounds, most were enriched in metabolism-related categories (Figure 7E). Further analyses integrating the LINCS L1000 database and a traditional Chinese medicine (TCM) compound library were conducted, and the top 25 and bottom 25 ranked compounds were selected for subsequent evaluation. Clustering analysis revealed transcriptional response patterns between the two compound groups (Figure 7F). Visualization at single-cell resolution further demonstrated marked heterogeneity in compound activity distributions across different cellular populations (Figure 7G).

By integrating drug response scores, transcriptional response patterns, and cellular distribution characteristics, six highly enriched compounds associated with hypertension-related molecular features were ultimately selected for subsequent functional validation, including three small-molecule drugs (CNX-774, PRT-062607, and Sinensetin) and three TCM–derived compounds (Pseudoginsenoside-F11, epicatechin gallate, and isorhynchophylline) (Figure 7F,G).

To further evaluate the anticancer activity and safety profiles of the candidate compounds, in vitro drug sensitivity assays were performed using normal renal tubular epithelial cells (HK-2) and renal cancer cell lines (ACHN and 786-O). As shown in Figure 7H,I, PRT-062607 exhibited lower IC50 value in HK-2 cells than in renal cancer cells, indicating substantial cytotoxicity toward normal renal epithelial cells and poor tumor selectivity. In contrast, isorhynchophylline and sinensetin exhibited only weak or negligible inhibitory effects in renal cancer cells; therefore, they were excluded from further investigation.

Further analysis revealed that CNX-774, pseudoginsenoside-F11, and epicatechin gallate (ECG) exhibited selective cytotoxic effects against renal tumor cells. Among these compounds, CNX-774 showed strong inhibitory activity in 786-O cells, with markedly lower IC50 value than those observed HK-2 cells. Notably, both pseudoginsenoside-F11 and ECG exhibited pronounced differences in IC50 values between normal renal tubular epithelial cells and renal cancer cells. In particular, ECG exhibited significantly lower IC50 values in ACHN and 786-O cells than in HK-2 cells, indicating potent antitumor activity with relatively low cytotoxicity toward normal cells suggesting a favorable therapeutic window and safety profile. Therefore, pseudoginsenoside-F11 and ECG were considered promising candidates for further investigation.

Collectively, this study established a single-cell transcriptomics-based drug screening framework and identified several candidate compounds with potential anti-ccRCC activity and antihypertensive therapeutic potential through integrated computational prediction and in vitro validation, thereby providing new perspectives for precision treatment strategies in patients with ccRCC complicated by hypertension.

## 4. Discussion

Clear cell renal cell carcinoma (ccRCC) is a multifactorial disease influenced by genetic alterations, environmental exposures, and systemic metabolic factors. Among these factors, hypertension is not only one of the most common comorbidities in ccRCC but also an established risk factor for renal carcinogenesis. Persistent hypertension can induce renal ischemia, oxidative stress, chronic inflammation, and activation of the renin–angiotensin–aldosterone system (RAAS), thereby promoting a hypoxic and pro-inflammatory microenvironment favorable for tumor initiation and progression [37,38,39]. Although previous studies have implicated pathways such as HIF-1α, VEGF, and angiotensin II signaling in this process, the molecular networks linking hypertension and ccRCC remain insufficiently characterized [40,41,42].

In the present study, integrated transcriptomic analysis identified four stable hypertension-related hub genes—*SCNN1G*, *CASR*, *KCNJ1*, and *WNK4*—which were primarily enriched in ion transport, electrolyte homeostasis, and aldosterone-associated signaling pathways. These findings suggest that dysregulated renal ion homeostasis may be associated with the molecular relationship between hypertension and ccRCC. Furthermore, diagnostic models constructed using LASSO and Random Forest algorithms exhibited strong predictive performance, supporting the robustness of the identified hypertension-related molecular signatures and their potential for further diagnostic evaluation. GSVA additionally revealed that elevated hypertension-related transcriptional activity was associated with poorer overall survival, suggesting a potential prognostic relevance of these molecular alterations in ccRCC.

Among the identified hub genes, *SCNN1G* encodes the γ-subunit of the epithelial sodium channel (ENaC), which is essential for sodium reabsorption and electrolyte balance. Previous studies have suggested that abnormal ENaC activity can influence membrane potential, inflammatory signaling, and metabolic reprogramming [43,44]. Consistently, *SCNN1G* was significantly downregulated in ccRCC, and its silencing promoted RCC cell proliferation, migration, and invasion, suggesting a potential tumor-suppressive role. *CASR*, *KCNJ1*, and *WNK4* are likewise closely involved in renal ion transport and blood pressure regulation. Dysregulation of these genes may disturb ionic and metabolic homeostasis, thereby facilitating hypoxia, angiogenesis, and tumor microenvironment remodelling [45,46,47]. Collectively, these results support the concept that hypertension-related ion transport abnormalities may contribute to ccRCC progression through coordinated metabolic and microenvironmental reprogramming.

Spatial transcriptomics analysis further demonstrated that hypertension-related molecular signatures were predominantly enriched in stromal and immune-infiltrated regions rather than in tumor cores, suggesting that these alterations may be more closely associated with tumor microenvironment remodeling. Given the recognized heterogeneity of ccRCC, such spatial distribution patterns support the hypothesis that dysregulated ion homeostasis may influence immune regulation, inflammatory responses, and metabolic adaptation within the tumor ecosystem [48,49,50,51].

In addition, single-cell transcriptomic analysis enabled the construction of a cell-state–resolved drug screening framework. Compared with conventional bulk RNA-seq–based approaches, this strategy more precisely captured transcriptional heterogeneity and differential drug sensitivity across cellular subpopulations [52]. This strategy is consistent with recent advances in computational herbal medicine research, where integrative databases and transcriptome-guided approaches have facilitated the systematic discovery of bioactive compounds and therapeutic candidates [53]. Notably, pseudoginsenoside-F11 and epicatechin gallate (ECG) exhibited selective cytotoxicity toward renal cancer cells with relatively low toxicity in normal renal tubular epithelial cells, suggesting a favorable therapeutic window. Previous studies have reported antioxidant, anti-inflammatory, and anti-tumor activities for these compounds, supporting their potential translational value [54,55,56,57]. Interestingly, ECG has also been reported to exert antihypertensive effects through activation of the KCNQ5 potassium channel, thereby promoting vascular relaxation and reducing arterial tension [58]. These findings further support the potential translational value of ECG in the comorbid context of ccRCC and hypertension.

Several limitations should be acknowledged. First, due to the lack of publicly available datasets containing both hypertension and ccRCC information, we were unable to directly validate the identified hypertension-related molecular signatures in independent cohorts. Second, this study was based mainly on retrospective public datasets. The absence of detailed hypertension-related clinical information and the limited availability of large independent cohorts restricted further clinical stratification analyses and external validation of the machine learning models. Third, the spatial transcriptomic analysis was performed on a limited number of samples, and more comprehensive integration with single-cell transcriptomic data is needed to better characterize cell-type-specific molecular features. Finally, the candidate therapeutic compounds identified in this study were based on computational prediction, and their underlying mechanisms and therapeutic efficacy require further experimental validation. Future studies integrating multi-transcriptomics profiling, clinical hypertension phenotypes, and functional experiments are needed to further validate these findings and clarify their potential clinical relevance in ccRCC [59,60,61].

In summary, this study systematically characterized hypertension-related molecular networks in ccRCC and identifies key hub genes, including *SCNN1G*, *CASR*, *KCNJ1*, and *WNK4*, as candidate hub genes associated with hypertension-related molecular alterations in ccRCC. By integrating bulk transcriptomic analysis with machine learning for biomarker identification, followed by spatial transcriptomic characterization, drug-response prediction, and experimental validation, this study establishes a comprehensive analytical framework for investigating hypertension-associated ccRCC. These findings provide additional insights into the potential molecular association between hypertension and ccRCC and identify candidate biomarkers and therapeutic compounds for future mechanistic and translational studies.

## 5. Conclusions

This study identified *SCNN1G*, *CASR*, *KCNJ1*, and *WNK4* as key hypertension-related hub genes in ccRCC and suggested that ion homeostasis dysregulation may associated with ccRCC progression. Integrated spatial and single-cell analyses further prioritized pseudoginsenoside-F11 and ECG as candidate therapeutic compounds and highlighted a potential molecular associationbetween hypertension and ccRCC.

## Data Availability

All datasets analysed in this study are publicly available: GeneCards (hypertension-related genes), UCSC Xena (TCGA-KIRC expression and clinical data), and GEO (GSE53757 and GSE210041). The analysis scripts (R code) and key intermediate files required to reproduce the main figures are provided as Supplementary Files. Data will be made available on reasonable request.

## Supplementary Materials

The following supporting information can be downloaded at: https://www.mdpi.com/article/10.3390/cancers18142250/s1, Figure S1: PPI network constructed within the DEHRGs; Figure S2: External validation of hub gene expression and diagnostic performance of clinical variables; Figure S3: Western blot analysis of SCNN1G protein expression in the human renal cancer cell lines 786-O and ACHN. β-Actin was used as the loading control; Table S1: Median expression values and interquartile ranges (IQRs) of the four hub genes in both tumor and solid tissue normal.

## Abbreviations

The following abbreviations are used in this manuscript:

AUC	area under the curve
ccRCC	clear cell renal cell carcinoma
CNV	copy number variation
DCA	decision curve analysis
DEGs	differentially expressed genes
DEHRGs	differentially expressed hypertension-related genes
ECG	epicatechin gallate
GEO	Gene Expression Omnibus
GO	Gene Ontology
GSVA	gene set variation analysis
HRGs	hypertension-related genes
IVW	inverse-variance weighted
KEGG	Kyoto Encyclopaedia of Genes and Genomes
LASSO	least absolute shrinkage and selection operator
MDA	mean decrease in accuracy
MDG	mean decrease in Gini
MR	Mendelian randomization
PPI	Protein–protein interaction
RAAS	renin–angiotensin–aldosterone system
RCC	renal cell carcinoma
RCTD	Robust Cell Type Decomposition
RF	random forest
ROC	receiver operating characteristic
TCGA	The Cancer Genome Atlas
TCM	traditional Chinese medicine
TOM	topological matrix
WGCNA	weighted gene co-expression network analysis
