# Supplementary figures and images for: Integrative Multi-Transcriptomic Uncovers Actionable Signatures and Drug Repurposing Candidates for ccRCC–Hypertension Comorbidity
Source: Cancers (Basel). 2026 Jul 14;18(14):2250. doi: 10.3390/cancers18142250 (PMC13406899; doi:10.3390/cancers18142250)

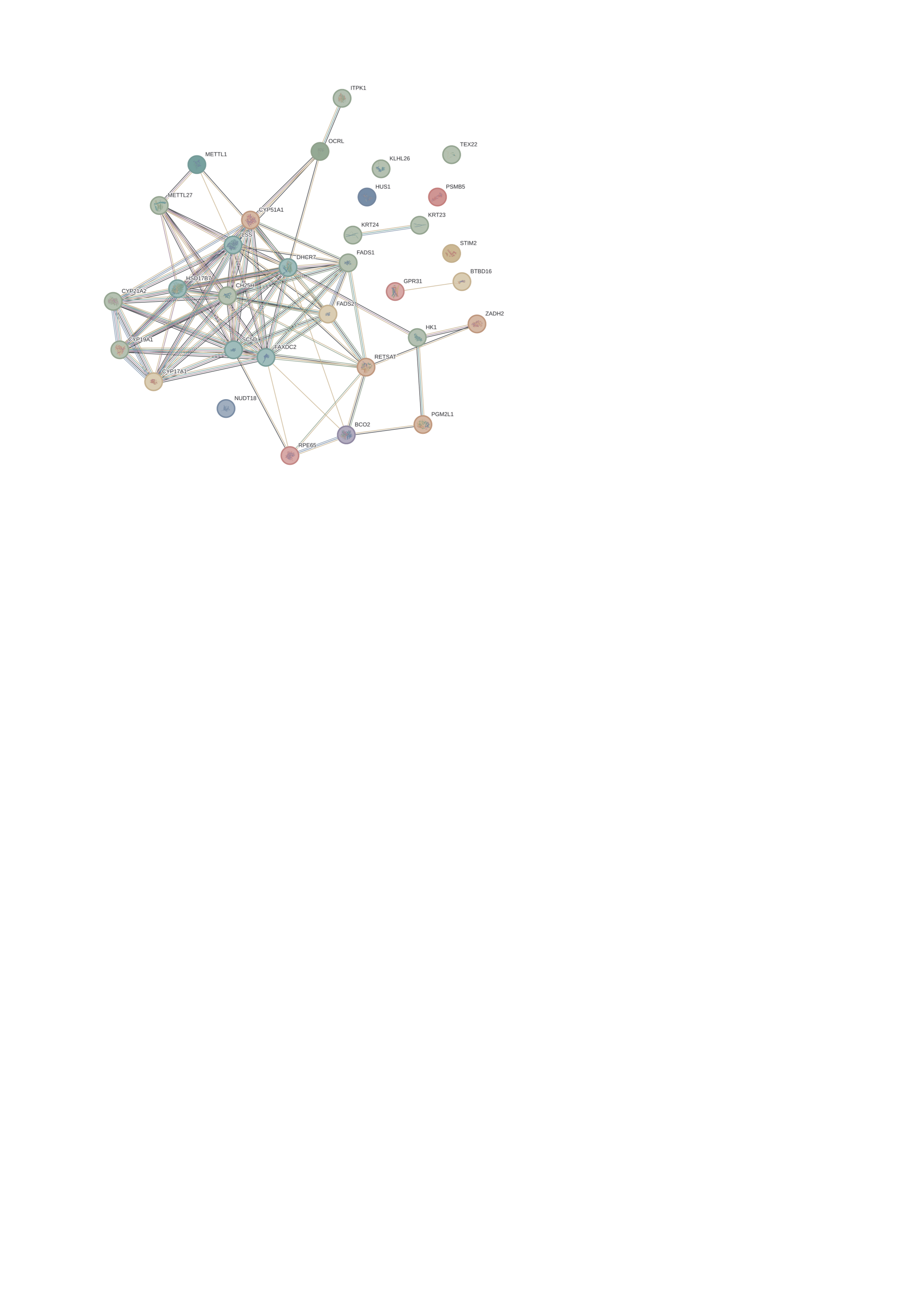

Supplement: Supplementary file 1 [file cancers-18-02250-s001.zip › Figure S1.png]

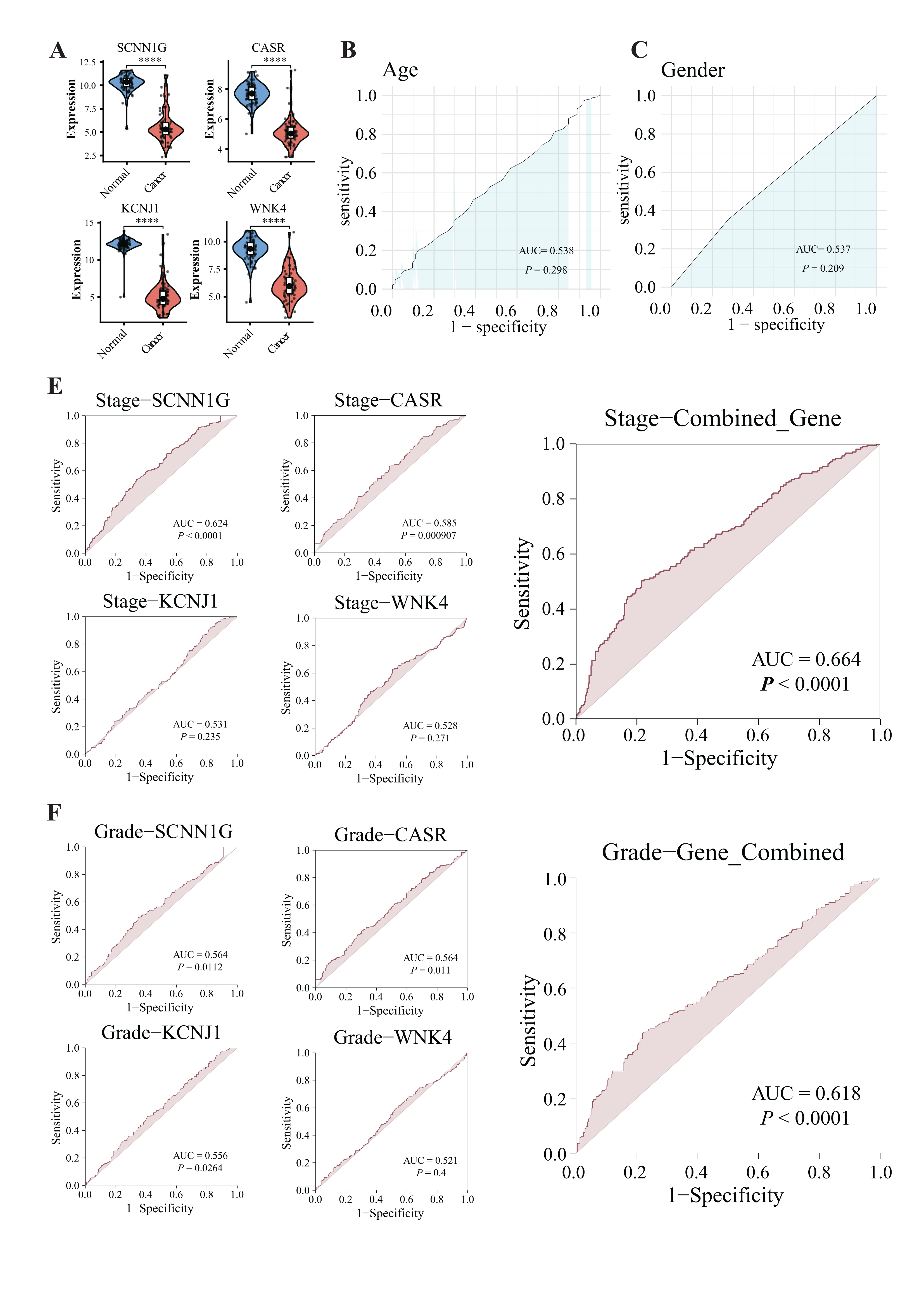

Supplement: Supplementary file 1 [file cancers-18-02250-s001.zip › Figure S2.png]

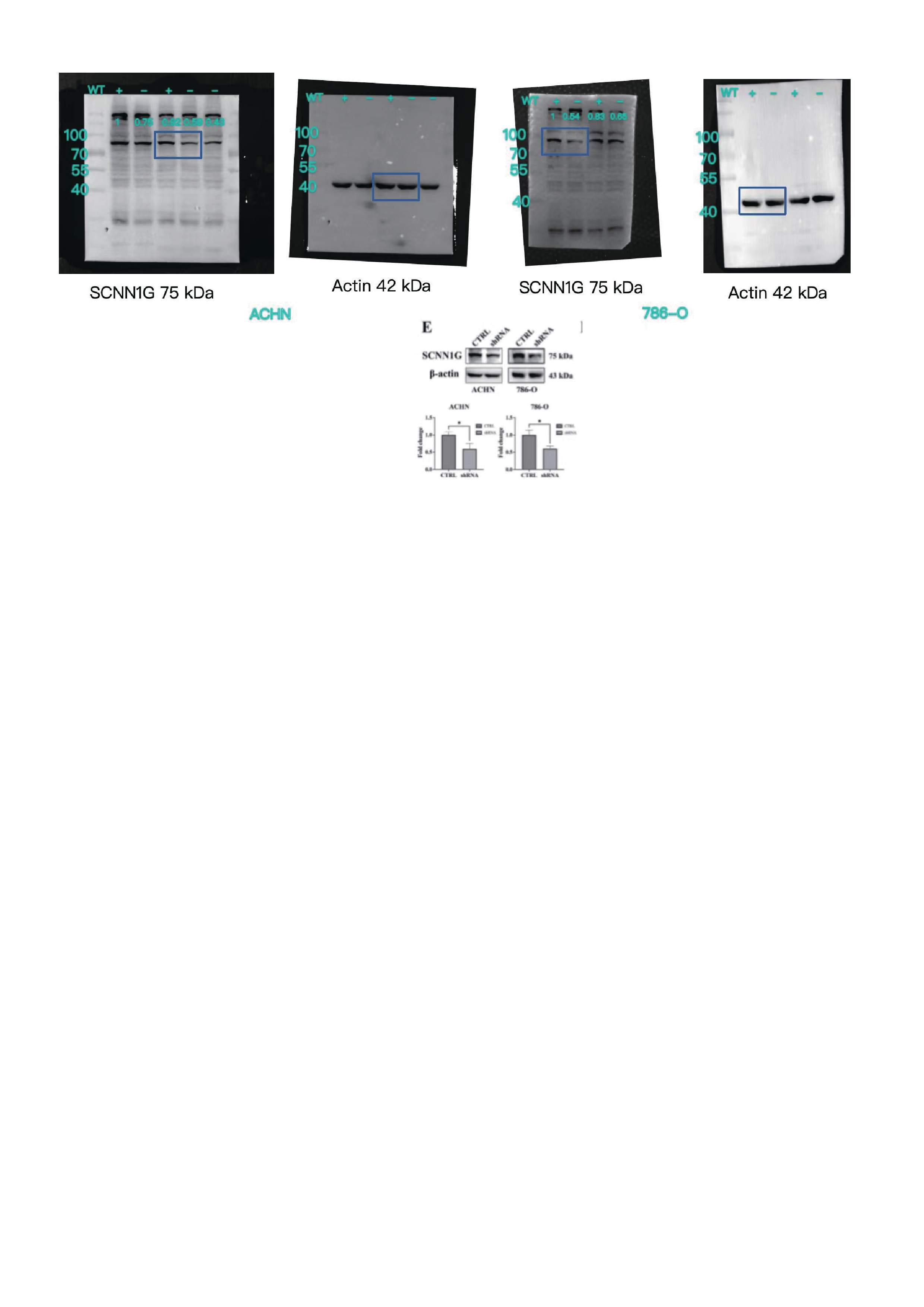

Supplement: Supplementary file 1 [file cancers-18-02250-s001.zip › Figure S3.png]
